# A randomized phase III trial of adjuvant chemotherapy with irinotecan, leucovorin and fluorouracil versus leucovorin and fluorouracil for stage II and III colon cancer: A Hellenic Cooperative Oncology Group study

**DOI:** 10.1186/1741-7015-9-10

**Published:** 2011-01-31

**Authors:** Christos A Papadimitriou, Pavlos Papakostas, Maria Karina, Lia Malettou, Meletios A Dimopoulos, George Pentheroudakis, Epaminontas Samantas, Aristotelis Bamias, Dimosthenis Miliaras, George Basdanis, Nikolaos Xiros, George Klouvas, Dimitrios Bafaloukos, Georgia Kafiri, Irene Papaspirou, Dimitrios Pectasides, Charisios Karanikiotis, Theofanis Economopoulos, Ioannis Efstratiou, Ippokratis Korantzis, Nikolaos Pisanidis, Thomas Makatsoris, Fotini Matsiakou, Gerasimos Aravantinos, Haralabos P Kalofonos, George Fountzilas

**Affiliations:** 1 Department of Clinical Therapeutics, "Alexandra" Hospital, University of Athens School of Medicine, Athens, Greece; 2 Oncology Department, "Ippokration" Hospital, Athens, Greece; 3 Department of Medical Oncology; "Papageorgiou" Hospital, Aristotle University of Thessaloniki School of Medicine, Thessaloniki, Greece; 4 Department of Biostatistics, Hellenic Cooperative Oncology Group Data Office, Athens, Greece; 5 Department of Medical Oncology, Ioannina University Hospital, Ioannina, Greece; 6 Third Department of Medical Oncology, "Agii Anargiri" Hospital, Athens, Greece; 7 Laboratory of Histology and Embryology, Aristotle University of Thessaloniki School of Medicine, Thessaloniki, Greece; 8 1st Propaedeutic Department of Surgery, "AHEPA" Hospital, Aristotle University of Thessaloniki School of Medicine, Thessaloniki, Greece; 9 2nd Propaedeutic Department of Internal Medicine, Oncology Section, University General Hospital "Attikon", Athens, Greece; 10 2nd Department of Medical Oncology, "Metropolitan" Hospital, Athens, Greece; 11 1st Department of Medical Oncology, "Metropolitan" Hospital, Athens, Greece; 12 Department of Pathology, "Ippokration" Hospital, Athens, Greece; 13 Histopathology Department, "Alexandra" Hospital, Athens, Greece; 14 424 Army General Hospital, Thessaloniki, Greece; 15 Department of Pathology, "Papageorgiou" Hospital, Thessaloniki, Greece; 16 Department of Medical Oncology, IKA Hospital, Thessaloniki, Greece; 17 Division of Oncology, Department of Medicine, University Hospital of Patras, Rion, Greece

## Abstract

**Background:**

Colon cancer is a public health problem worldwide. Adjuvant chemotherapy after surgical resection for stage III colon cancer has been shown to improve both progression-free and overall survival, and is currently recommended as standard therapy. However, its value for patients with stage II disease remains controversial. When this study was designed 5-fluorouracil (5FU) plus leucovorin (LV) was standard adjuvant treatment for colon cancer. Irinotecan (CPT-11) is a topoisomerase I inhibitor with activity in metastatic disease. In this multicenter adjuvant phase III trial, we evaluated the addition of irinotecan to weekly 5FU plus LV in patients with stage II or III colon cancer.

**Methods:**

The study included 873 eligible patients. The treatment consisted of weekly administration of irinotecan 80 mg/m^2 ^intravenously (IV), LV 200 mg/m^2 ^and 5FU 450 mg/m^2 ^bolus (Arm A) versus LV 200 mg/m^2 ^and 5FU 500 mg/m^2 ^IV bolus (Arm B). In Arm A, treatments were administered weekly for four consecutive weeks, followed by a two-week rest, for a total of six cycles, while in Arm B treatments were administered weekly for six consecutive weeks, followed by a two-week rest, for a total of four cycles. The primary end-point was disease-free survival (DFS) at three years.

**Results:**

The probability of overall survival (OS) at three years was 0.88 for patients in Arm A and 0.86 for those in Arm B, while the five-year OS probability was 0.78 and 0.76 for patients in Arm A and Arm B, respectively (P = 0.436). Furthermore, the probability of DFS at three years was 0.78 and 0.76 for patients in Arm A and Arm B, respectively (*P *= 0.334). With the exception of leucopenia and neutropenia, which were higher in patients in Arm A, there were no significant differences in Grades 3 and 4 toxicities between the two regimens. The most frequently recorded Grade 3/4 toxicity was diarrhea in both treatment arms.

**Conclusions:**

Irinotecan added to weekly bolus 5FU plus LV did not result in improvement in disease-free or overall survival in stage II or III colon cancer, but did increase toxicity.

**Trial registration:**

Australian New Zealand Clinical Trials Registry: ACTRN12610000148077

## Background

Colorectal cancer is one of the world's most common malignancies. After lung cancer, it is the most frequent cause of cancer-related mortality in the Western World [1]. Despite curative surgery in those presenting early, the risk of recurrence is significantly high with a mortality rate still close to 40%. Hence, much work has been done in search of effective adjuvant therapy for the eradication of disseminated micrometastases. In colon cancer, chemotherapy is the principal adjuvant therapy and the addition of radiotherapy to chemotherapy has not been shown to improve outcome [2]. In the absence of any further treatment after resection of the primary tumor, five-year survival rates are principally determined by the stage of the tumor at the time of resection. Studies performed in the late 1980s demonstrated that adjuvant fluorouracil (5FU) plus levamisole improved survival in patients with a resected stage III colon cancer. Further studies performed in the mid-1990s established 5FU plus leucovorin (LV) administered for approximately six months as a standard postoperative treatment. The therapeutic potential of systemic treatments for colorectal cancer has expanded rapidly during the past 10 years, with the introduction of oral fluoropyrimidines, oxaliplatin, and irinotecan [3-6].

Irinotecan is a topoisomerase I inhibitor with activity in metastatic colorectal cancer, alone or in combination with other agents [7,8], in both first- and second-line treatment of metastatic disease [9-13]. Cunningham *et al. *[14] randomized, in a 2:1 ratio, patients with metastatic colorectal cancer, which had progressed within six months of treatment with 5FU to receive either irinotecan with supportive care or supportive care alone and demonstrated a one-year survival rate 2.5 times greater for the irinotecan group than that achieved with best supportive care alone. Two further randomized trials of first-line therapy using irinotecan with or without 5FU plus LV demonstrated statistically significant survival advantages for the combination regimen [15,16]. As a result, the weekly irinotecan plus 5FU plus LV regimen became standard for first-line metastatic colorectal cancer. The improvements in response rate, progression-free survival (PFS), and overall survival (OS) achieved with the incorporation of irinotecan in the systemic treatment of patients with metastatic colorectal cancer encouraged its testing in the adjuvant setting, especially in patients with stage III disease. We performed a randomized controlled trial of the combination of weekly irinotecan plus LV plus 5FU *versus *a standard weekly schedule 5FU plus LV in the adjuvant setting following curative resection of stage II and III colon cancer.

## Methods

### Patient selection

All eligible patients had histologically confirmed colon adenocarcinoma and underwent complete surgery for stages B2 and C disease with neither gross nor microscopic evidence of residual tumor. The patients entered the study within three to six weeks after surgery; had not received any prior chemotherapy; were aged at least 18 years; had a World Health Organization (WHO) performance status ≤2; and should have no history of other malignancies except of adequately treated carcinoma *in situ *of the uterine cervix, or curatively treated non-melanomatous skin cancer or serious illness that would preclude protocol chemotherapy. Other laboratory eligibility requirements included absolute neutrophil count ≥1,500/μl, platelet count ≥100,000/μl, serum creatinine <1.5 mg/dl, and alanine transaminase (ALT) and aspartate aminotransferase (AST) levels less than twice the institutional upper limit of normal. Non eligibility criteria were histology other than adenocarcinoma, incomplete resection, myocardial infarction within the last six months or uncontrolled coronary insufficiency, inflammatory intestinal disease, and pregnant or nursing women. The clinical protocol and collateral translational studies were approved by the Hellenic Cooperative Oncology Group (HeCOG) Protocol Review Committee. The study was conducted in accordance with the ethical principles of the Declaration of Helsinki and Good Clinical Practice. Our study was also registered at the Australian New Zealand Clinical Trials Registry (ACTRN12610000148077). Before randomization all patients provided written informed consent and eligibility was confirmed by a protocol-specific checklist.

### Evaluation

Before study entry, all patients underwent a complete physical examination, assessment of performance status, complete blood cell (CBC) count and differential, liver and kidney function tests, serum carcinoembryonic antigen (CEA) and cancer antigen (CA) 19-9, urinalysis, electrocardiogram (ECG), pelvic and abdominal computed tomography, and chest X-ray with computed tomography of the chest when clinically indicated. During treatment, clinical evaluation and CBC were performed every week. Furthermore, biochemistry laboratory evaluations were performed every two weeks until the end of the treatment, and then every three months thereafter. Abdominal and pelvic CT scans and chest-X-ray were performed at the end of the study, and then every six months thereafter. However, CT scans were repeated earlier whenever clinically indicated depending on the discretion of the investigator. Endoscopy was performed annually [12,17].

### Treatment

Eligible and registered patients were randomly assigned using central registration to receive either irinotecan plus LV plus 5FU (Arm A) or LV plus 5FU (Arm B). Chemotherapy in Arm A consisted of irinotecan 80 mg/m^2 ^in 250 ml normal saline, as a 90-minute intravenous (IV) infusion, followed by LV 200 mg/m^2 ^in 500 ml normal saline, IV over two hours, and 5FU 450 mg/m^2^, as IV rapid administration, 60 minutes after initiation of LV infusion. Treatments were administered weekly for four consecutive weeks, followed by a two-week rest, for a total of six cycles. The LV plus 5FU group (Arm B) received chemotherapy consisting of LV 200 mg/m^2 ^by IV injection over two hours, with a bolus of 5FU 500 mg/m^2 ^administered by IV injection at 60 minutes after initiation of LV. Treatments were administered weekly for six consecutive weeks, followed by a two-week rest, for a total of four cycles.

### Dose modification

Toxicities were graded using the WHO common criteria. Dose adjustments of the drugs or treatment delays were decided according to the worst toxicity grade at preceding cycle. The dose modification was determined according to the body system showing the greatest toxicity. Chemotherapy was interrupted if Grade more than 2 toxicity was encountered and was restarted when toxicity had resolved to ≤Grade 1. In case of diarrhea, the patients underwent supportive care and also intensive treatment with loperamide and were hospitalized if necessary. The doses of irinotecan and 5FU were reduced by 20% or 30% in the case of Grades 2 and 3 diarrhea, respectively. In the presence of Grades 1, 2 and 3 hematological toxicity, treatment was delayed for at least one week until hematological recovery without dose reductions in further infusions, while in the case of Grade 4 hematological toxicity, irinotecan as well as the 5FU dose was reduced by 20% in all the subsequent courses.

There was no re-escalation for patients experiencing bone marrow or gastrointestinal toxicity requiring dose modification. In the case of hand-foot syndrome (Grade 3 or 4), only the dose of 5FU was to be reduced by 20%. In the case of angina or myocardial infarction the treatment would be ceased. Furthermore, for Grade 3 or 4 mucositis, there was a possibility for restarting the treatment after one week delay, if toxicity had resolved to ≤Grade 1 and the dose of irinotecan and 5FU was to be reduced by 20%. In the presence of any Grade 4 toxicity, except for gastrointestinal or hematological toxicity, the patients were withdrawn from the study. No prophylactic treatment was permitted for diarrhea. Specific guidelines for curative treatment of delayed diarrhea were provided, which recommended loperamide 2 mg every 2 h for 12 h after the last loose stool, for a maximum of 48 consecutive hours. If diarrhea was not controlled after 48 h of non-stop loperamide intake, or if the patient was dehydrated, loperamide was stopped and the patient was hospitalized for intravenous fluids.

### Statistical analysis

For a two-sided test at the 5% level of significance and a power of 80%, the number of patients required to detect a difference between the two treatment arms within 5% (± 2.5%) to the baseline rate of 80% in DFS at the three-year time point [18] was 870 patients. Taking into account a 3% withdrawal, 900 patients (450 per group) were needed to enter the study with an accrual rate of 170 patients per year and a corresponding maximum study duration of 9.9 years. An interim analysis based on the O'Brien Fleming boundary values was performed when half (50%) of the endpoints (161 relapses) had been reached. The study would be ended prematurely if either a significant difference was detected or the alternative was rejected at the interim analysis. No significant difference in DFS rate was detected (*P *= 0.112) and the study was continued to completion.

Overall survival (OS) was measured from the date of randomization until death from any cause. Surviving patients were censored at the date of last contact. DFS was measured from the date of randomization until recurrence of tumor or secondary malignancy or death from disease without relapse. "Secondary malignancy" includes any cancer and not just another colorectal cancer. Death from disease without relapse means death without documented progression of the disease, which includes deaths from any cause.

Continuous variables were presented as medians with the corresponding range and categorical variables as frequencies with the respective percentages. The Fisher's exact test and the non-parametric Mann-Whitney were used for comparing patient and tumor characteristics. Time to event distributions were estimated using Kaplan-Meier curves and compared using the log-rank test. The Cox proportional hazards models were used to assess the relationship of OS and DFS with various clinical and histological variables. The backward selection procedure with removal criterion *P *>0.10 based on Likelihood ratio test was performed to identify significant variables among the following: treatment group (Arm A *versus *Arm B), age, histology grade (I *versus *II to III), Dukes stage (B *versus *C), number of examined lymph nodes, number of involved lymph nodes, obstruction (no *versus *yes), perforation (no *versus *yes), primary site (right colon *versus *left colon *versus *sigmoid). All statistical tests were two-sided and performed at a significance level of 0.05. The SPSS software was used for statistical analysis (SPSS for Windows, version 18.0 PASW Statistics, IBM, Chicago, IL). Analysis was carried out following the modified intention-to-treat principle, that is, including all eligible patients.

## Results

**Figure 1:**
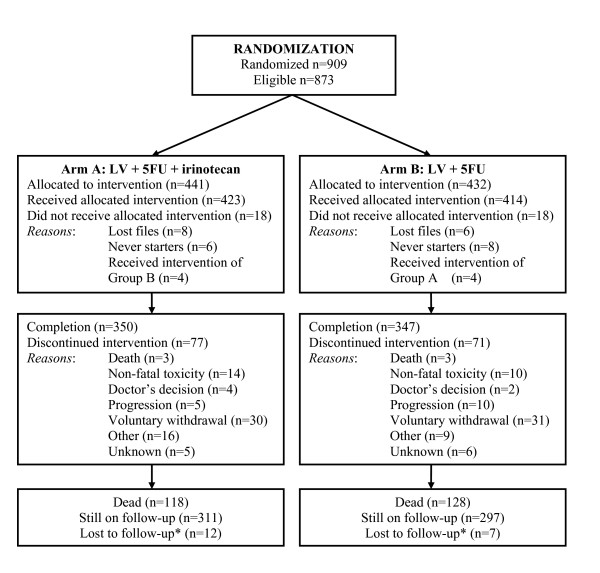
**Outline of the study**. *When followed-up for less than four years

Between January 1999 and September 2004, 909 patients were randomly assigned to irinotecan plus 5FU plus LV or to 5FU plus LV. Thirty-six patients (4%) were ineligible: 15 had metastatic disease, 11 had rectal cancer, 5 had non radical surgery, 3 had a history of other malignancy, 1 had positive margins, and 1 had ovarian adenocarcinoma. The outline of the study is shown in Figure 1. For 14 patients medical records were incomplete at the time of the analysis (8 from Arm A and 6 from Arm B). Furthermore, six patients in Arm A and eight patients in Arm B never started on chemotherapy; these patients were included in the efficacy analysis according to the intent-to-treat method, but were excluded from the toxicity and treatment characteristics analyses. Four patients randomized to Arm A received Arm B treatment and *vice versa*. Patient characteristics were well balanced for age, gender, T and N stage, median number of nodes reported, and the presence of perforation or obstruction (Table 1).

**Table 1:** Patient characteristics

Characteristic	Arm A: IRI+LV+5FU N = 441 (%)	Arm B: LV+5FU N = 432 (%)	*P*
**Age (years)**			0.336
Median	65	65	
Range	26 to 79	24 to 79	
**Sex**			0.892
Female	206 (47)	199 (46)	
Male	235 (53)	233 (54)	
**Stage (Dukes)**			0.946
B	214 (48)	211 (49)	
C	227 (52)	221 (51)	
**Primary tumor (T)**			0.891
T1 and T2	29 (6)	27 (6)	
T3 and T4	405 (92)	399 (93)	
Unknown	7 (2)	6 (1)	
**Regional lymph nodes (N)**			0.350
N0	210 (48)	208 (48)	
N1	153 (35)	134 (31)	
N2	70 (16)	82 (19)	
Unknown	8 (2)	8 (2)	
**Histology grade**			0.048
I	40 (9)	47 (11)	
II	334 (76)	296 (68)	
III	62 (14)	84 (20)	
Unknown	5 (1)	5 (1)	
**Number of lymph nodes examined**			0.354
Median	14	14	
Range	0 to 96	0 to 70	
Number of lymph nodes involved*			0.271
Median	2	3	
Range	1 to 23	1 to 27	
**Site of disease**			0.640
Cecum	88 (20)	95 (22)	
Ascending colon	67 (15)	67 (16)	
Transverse colon	29 (7)	30 (7)	
Descending colon	36 (8)	44 (10)	
Sigmoid	218 (49)	194 (45)	
Unknown	4 (1)	2 (0.5)	
**Perforation**			0.897
Yes	406 (92)	398 (92)	
No	33 (7)	31 (7)	
Unknown	2 (1)	3 (1)	
**Obstruction**			0.277
Yes	386 (88)	365 (84)	
No	54 (12)	64 (15)	
Unknown	1 (0.2)	3 (1)	

*Only for stage C disease			

Out of the 873 eligible patients, 148 discontinued treatment, 77 (18%) patients in Arm A and 71 (17%) in Arm B, respectively. The most common reason for treatment discontinuation was voluntary withdrawal (30 patients in Arm A and 31 patients in Arm B). Additional reasons for treatment discontinuation were toxicity (14 patients in Arm A and 10 patients in Arm B), disease progression (5 *versus *10), death (3 *versus *3), physician's decision (4 *versus *2) and others (16 *versus *9). In all, 350 (79%) patients randomized in Arm A and 347 (80%) in Arm B completed treatment.

### Efficacy

**Figure 2:**
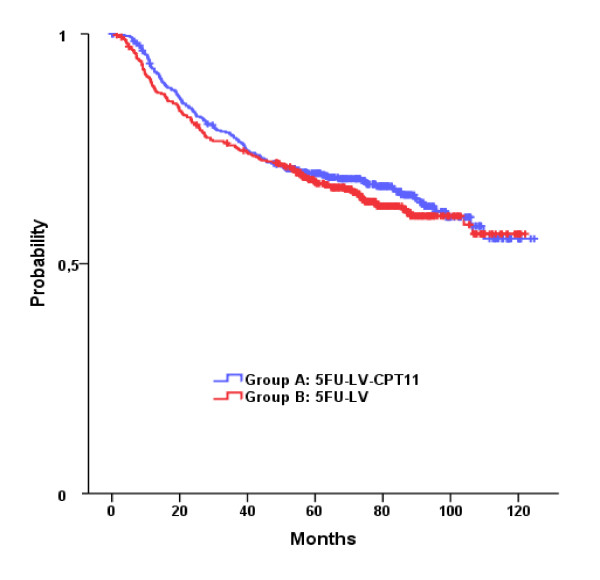
**Kaplan-Meier curves for the DFS in the two groups**.

**Figure 3:**
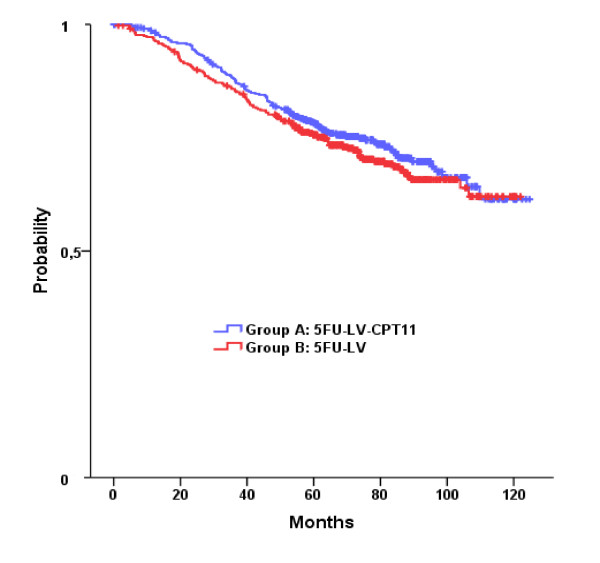
**Kaplan-Meier curves for the OS in the two groups**.

The median follow-up time was 77.4 months (range, 0.1 to 124.7). There were no significant differences between irinotecan plus LV plus 5FU and LV plus 5FU in OS and DFS (Table 2). Survival curves are presented in Figures 2 and 3. The probability of OS at three years was 0.88 for patients in Arm A and 0.86 for those in Arm B, while the five-year OS probability was 0.78 and 0.76 for patients in Arm A and Arm B, respectively. Furthermore, the probability of DFS at three years was 0.78 and 0.76 for patients in Arm A and Arm B, respectively. Five-year DFS probability was 0.70 for Arm A and 0.68 for Arm B.

**Table 2:** Disease-free survival and overall survival

	Arm A IRI+LV+5FU	Arm B LV+5FU	*P *(log-rank)
**Disease-free survival (DFS)**			0.436
Progressions, *n (%)*	110 (24.9)	112 (25.9)	
Median (months)	Not reached yet	Not reached yet	
three-year DFS (%)	78	76	
five-year DFS (%)	70	68	
seven-year DFS (%)	66	63	
**Overall survival (OS)**			0.334
Deaths, *n (%)*	118 (26.8)	128 (29.6)	
Median (months)	Not reached yet	Not reached yet	
three-year OS (%)	88	86	
five-year OS (%)	78	76	
seven-year OS (%)	72	69	

When patients were analyzed by stage (Table 3), those with Dukes B disease had an OS probability at three years of 0.93 in Arm A and 0.89 in Arm B, while their five-year OS probability was 0.86 and 0.83 in Arms A and B, respectively. Furthermore, the probability of DFS at three years was 0.86 and 0.83 for patients in Arms A and B, respectively. The three-year OS probability for patients with stage C disease was 0.79 in Arm A and 0.82 for those in Arm B, while their five-year OS probability was 0.71 and 0.68 in Arms A and B, respectively. DFS probability at three years was 0.69 and 0.68 for patients in Arms A and B, respectively. Only 24 (2.7%) patients out of 873 who were analyzed developed secondary malignancy (10 in arm A and 14 in arm B). Our results do not change if we use the definition of DFS as used in the MOSAIC trial (that is, not including secondary malignancies) [19].

**Table 3:** Disease-free survival and overall survival stratified by stage

	Arm A IRI+LV+5FU	Arm B LV+5FU	*P *(log-rank)
**Stage B (n = 425)**			

**Disease-free survival (DFS)**			0.561
Progressions, *n (%)*	36 (16.8)	35 (16.6)	
Median (months)	Not reached yet	Not reached yet	
three-year DFS (%)	86	83	
five-year DFS (%)	79	78	
seven-year DFS (%)	75	73	
**Overall survival (OS)**			0.389
Deaths, *n (%)*	39 (18.2)	46 (21.8)	
Median (months)	Not reached yet	Not reached yet	
three-year OS (%)	93	89	
five-year OS (%)	86	83	
seven-year OS (%)	80	78	

**Stage C (n = 448)**			

**Disease-free survival (DFS)**			0.515
Progressions, *n (%)*	74 (32.6)	77 (34.8)	
Median (months)	106	104	
three-year DFS (%)	69	68	
five-year DFS (%)	60	57	
seven-year DFS (%)	57	52	
**Overall survival (OS)**			0.539
Deaths, *n (%)*	79 (34.8)	82 (37.1)	
Median (months)	Not reached yet	Not reached yet	
three-year OS (%)	83	83	
five-year OS (%)	71	68	
seven-year OS (%)	64	59	

The median relative dose intensity for 5FU was 0.97 (range 0.24 to 1.27) in Arm A and 0.86 (0.14 to 1.10) in Arm B (*P *< 0.001). The relative dose intensity for irinotecan in Arm A was 0.95 (range 0.21 to 1.27) (Table 4). In univariate analysis, the most important prognostic factors were age at diagnosis as a continuous variable hazard rate (HR) = 1.03, 95% CI 1.02 to 1.05, *P *< 0.001 for OS and HR = 1.02, 95% CI 1.01 to 1.03, *P *= 0.001 for DFS), stage C *versus *B (HR = 2.04, 95% CI 1.57 to 2.65, *P *< 0.001 for OS and HR = 2.06, 95% CI 1.63 to 2.60, *P *<0.001 for DFS), T3 and T4 *versus *T1 and T2 disease (HR = 2.72, 95% CI 1.28 to 5.77, *P *= 0.009 for OS and HR = 2.64, 95% CI 1.36 to 5.13, *P *= 0.004 for DFS), number of involved lymph nodes as continuous variable (HR = 1.10, 95% CI 1.08 to 1.13, *P *<0.001 for OS and HR = 1.10, 95% CI 1.08 to 1.13, *P *<0.001 for DFS), the presence or not of obstruction (HR = 1.35, 95% CI 0.97 to 1.88, *P *= 0.076 for OS and HR = 1.37, 95% CI 1.01 to 1.85, *P *= 0.041 for DFS), and the presence or not of perforation (HR = 1.46, 95% CI 0.95 to 2.52, *P *= 0.082 for OS and HR = 1.61, 95% CI 1.10 to 2.35, *P *= 0.013 for DFS).

**Table 4:** Dose intensity (DI)

	Arm A: IRI+LV+5FU N = 427	Arm B: LV+5FU N = 418	*P*
**DI of irinotecan (mg/m**^**2**^**/wk)**			
Median	50.5	NA*	
Range	11 to 68	NA	
**Relative DI of irinotecan**			
Median	0.95	NA	
Range	0.21 to 1.27	NA	
**DI of LV (mg/m**^**2**^**/wk)**			< 0.001
Median	129	131	
Range	32 to 165	21 to 279	
**Relative DI of LV**			
Median	0.96	0.87	
Range	0.24 to 1.24	0.14 to 1.86	
**DI of 5FU (mg/m**^**2**^**/wk)**			< 0.001
Median	291	322	
Range	73 to 380	54 to 411	
**Relative DI of 5FU**			
Median	0.97	0.86	
Range	0.24 to 1.27	0.14 to 1.10	

*Not applicable			

In the multivariate model, only age at diagnosis (HR = 1.03, 95% CI 1.02 to 1.05, *P *< 0.001), stage (C *versus *B) (HR = 1.41, 95% CI 1.04 to 1.92, *P *= 0.028) and number of involved lymph nodes (HR = 1.09, 95% CI 1.05 to 1.12, *P *< 0.001) maintained predictive significance for OS. Similarly, older age (HR = 1.02, 95% CI 1.01 to 1.03, *P *= 0.002), stage (C versus B) (HR = 1.44, 95% CI 1.10 to 1.90, *P *= 0.009), number of involved lymph nodes (HR = 1.09, 95% CI 1.05 to 1.12, *P *< 0.001) and the presence of obstruction (HR = 1.43, 95% CI 1.05 to 1.94, *P *= 0.022) were found to be significantly associated with poorer DFS.

### Toxicity

Serious adverse events associated with each treatment regimen are listed in Table 5. Regarding toxicity, patients were analyzed according to the treatment patients actually received. With the exception of leucopenia and neutropenia, which were higher in patients in Arm A (*P *= 0.037 and *P *< 0.001, respectively), there were no significant differences in Grades 3 and 4 toxicities between the two regimens. The most frequently recorded Grade 3/4 toxicity was diarrhea in both treatment arms, followed by neutropenia. Overall, 116 patients in group A (27%) experienced any severe toxicity versus 76 patients in group B (18%), *P *= 0.002. In 14 (3%) patients on Arm A and 10 (2%) patients on Arm B, treatment was stopped due to an adverse event. The unscheduled hospital admissions of patients due to treatment Grade 3 or 4 toxicity were 39 (9%) in Arm A and 47 (11%) in Arm B (*P *= 0.363). Granulocyte colony-stimulating factor (G-CSF) because of leucopenia/neutropenia was administered in 135 patients, 98 (23%) in Arm A and 37 (9%) in Arm B (*P *< 0.001). Of the 246 deaths that occurred during the follow-up period, 241 (98%) were due to colon cancer, 2 due to toxicity of the treatment (neutropenic sepsis) and 4 due to other causes such as pulmonary embolism (2 patients), acute myocardial infarction and CNS ischemia.

**Table 5:** Incidence of Grades 3 and 4 toxicities (treatment as administered)

	Arm A: IRI+LV+5FU (N = 427)		Arm B: LV+5FU (N = 418)	
	
	*n (%)*		*n (%)*	
	
	Grade 3	Grade 4	Grade 3	Grade 4
**Leukopenia**	10 (2)	0	2 (0.5)	0
**Neutropenia**	39 (9)	6 (1)	7 (2)	4 (1)
**Anemia**	2 (0.5)	0	0	0
**Thrombocytopenia**	2 (0.5)	0	0	0
**Diarrhea**	58 (14)	6 (1)	52 (12)	2 (0.5)
**Nausea/vomiting**	7 (2)	0	1 (0.2)	0
**Alopecia**	6 (1)	0	1 (0.2)	0
**Neutropenic fever**	4 (1)	0	1 (0.2)	0
**Constipation**	2 (0.5)	0	1 (0.2)	0
**Pain**	1 (0.2)	0	2 (0.5)	0
**Hepatotoxicity**	1 (0.2)	0	2 (0.5)	0
**Mucositis**	0	0	2 (0.5)	0
**Fatigue**	1 (0.2)	0	0	0
**Watery eye**	0	0	1 (0.2)	0
**Peripheral neuropathy**	0	0	1 (0.2)	0
**Skin**	0	0	1 (0.2)	0

## Discussion

This study was designed to assess the impact on the three-year disease-free survival of the addition of irinotecan to conventional adjuvant treatment with LV-modulated bolus 5FU, following curative resection of stage II or III colon cancer. Based on our experience with irinotecan's safety and efficacy [12] and on the encouraging results of several studies in patients with metastatic disease [9-14], it was anticipated that irinotecan would be an effective addition to adjuvant treatment program for colon cancer. Our study did not demonstrate a statistically significant difference in the three-year disease-free and overall survival between the study arms.

In agreement with our study, three large prospective randomized trials evaluating the addition of irinotecan to bolus or continuous infusion of 5FU and LV had failed to show a survival benefit in colon cancer adjuvant setting. In the CALGB 89803 trial, Saltz *et al. *compared conventional bolus 5FU plus LV with or without addition of irinotecan [20]. After resection of stage III colon cancer, 1,264 patients were assigned randomly to receive a conventional regimen (weekly LV 500 mg/m^2 ^plus 5FU 500 mg/m^2^, administered for six consecutive weeks followed by two weeks of rest, for four cycles) or the experimental IFL regimen (weekly irinotecan 125 mg/m^2 ^and LV 20 mg/m^2 ^plus 5FU 500 mg/m^2^, administered for four consecutive weeks followed by two weeks of rest, for five cycles). Lethal and nonlethal toxicity was significantly greater for IFL than for LV plus 5FU. On the other hand, no differences were found at three years for IFL compared with LV plus 5FU in the probability of overall survival, disease-free survival, or relapse-free survival; similarly, no differences were seen in five-year outcomes.

The negative results of CALGB 89803 are mirrored in two recently published European adjuvant trials of irinotecan plus continuous infusion 5FU (FOLFIRI), which failed to lengthen disease-free survival in colon cancer after surgical resection: PETACC-3 and ACCORD. More specifically, the PETACC-3 study investigated whether the addition of irinotecan to the de Gramont infusional 5FU and LV adjuvant regimen (LV5FU2) would improve DFS in patients with stage III colon cancer. The principal efficacy analysis was based on 2,094 treated patients, randomly allocated in the LV5FU2 strata. Severe gastrointestinal and hematologic toxicity was increased in patients receiving irinotecan [21]. The multicenter adjuvant phase III trial published by Ychou *et al. *evaluated the addition of irinotecan to LV5FU2 in colon cancer patients specifically at high risk of relapse [22]. This study randomly assigned 400 patients with either N1 tumors with obstruction/perforation or N2 tumors to LV5FU2, with or without irinotecan. Similarly, there was no evidence of improvement in DFS and OS in patients receiving irinotecan, while higher rates of Grades 3 and 4 neutropenia were observed.

Our study was designed to include both stage II and III patients in the analysis. Although historically many studies have combined the population of patients with both stage II and stage III disease, including some irinotecan adjuvant trials, the current trend is to perform separate clinical trials, since there are significant survival differences among patients with different T and N status. Some trials have been designed with a particular focus on high risk patients. Unfortunately, when high risk patients were evaluated, such as in the ACCORD study, as previously mentioned, irinotecan still did not provide significant benefit compared to 5FU and LV.

In the present study, we utilized a weekly IFL regimen based on our previous experience. More specifically, Kalofonos *et al. *[12] treated 55 patients with first-line chemotherapy for advanced disease with either irinotecan 80 mg/m^2 ^(7 patients) or 70 mg/m^2 ^(48 patients) plus LV 200 mg/m^2 ^and 5FU 450 mg/m^2^, weekly for six weeks followed by a two-week rest period. Treatment was continued for four cycles. Because of Grades 3 and 4 diarrhea in four of the first seven patients, the study was amended to reduce the starting dose of irinotecan from 80 to 70 mg/m^2 ^weekly. In another randomized phase II trial conducted by our Cooperative Group, 295 patients with metastatic colorectal cancer were randomized to receive as first-line chemotherapy either irinotecan 70 mg/m^2 ^plus LV and 5FU, as previously described, or oxaliplatin plus LV plus 5FU. Severe diarrhea occurred in 12.3% of patients of the irinotecan arm [17]. Hematological toxicity and gastrointestinal mucositis were a concern when the regimen in the experimental arm was designed because of our previous experience and data provided by Saltz *et al. *[16] on Grade 3 or 4 neutropenia and diarrhea in 53.8% and 22.7% of patients, respectively, who were treated with IFL for metastatic colon cancer. Therefore, we employed irinotecan at a relatively low dose of 80 mg/m^2 ^with LV 200 mg/m^2 ^and 5FU 450 mg/m^2^, weekly for four instead of six weeks, followed by the rest period. This dose of irinotecan was substantially reduced in comparison with the dose of 125 mg/m^2 ^which was utilized by Saltz *et al. *[20] in their adjuvant IFL regimen. On the other hand, treatment in Arm B was administered weekly for six consecutive weeks followed by the rest period that has resulted in combination with an increased by 10% 5FU dose in a statistically greater relative dose intensity for the fluoropyrimidine (0.97 *vs*. 0.86, *P *<0.001). However, these differences are unlikely to have any impact on efficacy.

With the exception of leucopenia and neutropenia, which were higher in patients in Group A, there were no other significant differences in Grades 3 and 4 toxicities between the two regimens. The most frequently recorded Grade 3/4 toxicity was diarrhea in both treatment arms, followed by neutropenia. More specifically, severe diarrhea was observed in 15% and 12.5% of patients in Group A and Group B, respectively, while the corresponding rates for patients treated with either IFL or LV plus 5FU in the CALGB 89803 study were 31% and 35%, respectively. On the other hand, Grade 3/4 neutropenia of the irinotecan plus LV plus 5FU arm was similar to that reported by Saltz *et al. *[20]. In this latter study, a higher incidence of treatment-related deaths has been reported with the IFL regimen compared to LV plus 5FU. However, in the present study this increased incidence was not observed in the experimental arm, possibly due to the dose level of irinotecan. Because of the higher incidence of severe neutropenia, more G-CSF was administered in patients who were allocated to irinotecan-based chemotherapy arm.

Based on the results of the present and three previous negative trials [20-22], irinotecan should not be used in the adjuvant setting, because it adds no benefit when combined with either bolus or continuous-infusion 5FU. The question of why irinotecan has failed to demonstrate an advantage in the adjuvant setting while oxaliplatin is clearly of benefit still remains unanswered. Possible explanations involve clinical and pharmacologic aspects. Although the two head-to-head comparisons of infusional 5FU, LV, and oxaliplatin (FOLFOX) and infusional 5FU, LV, and irinotecan (FOLFIRI) in metastatic disease [23,24] failed to show a significant difference in progression-free survival, each of these studies was remarkably underpowered to rule out a clinically meaningful difference between these regimens. Thus, the lack of adequately powered head-to-head comparisons between FOLFOX and FOLFIRI leaves open the possibility that FOLFOX may be superior to FOLFIRI [6].

In the PETACC-3 trial, the patients enrolled in the irinotecan arm experienced more dose reductions and more treatment discontinuation because of toxicity compared with the LV5FU2 arm. On the other hand, in this study after adjustment for imbalances in the TNM status between treatment groups, a multivariate analysis "rendered" a statistically significant DFS advantage in favor of the irinotecan arm (*P *= 0.021), while a highly significant relapse-free survival advantage for the same arm could be achieved (*P *= 0.009) [21]. Furthermore, the concept of individualized therapy based on prognostic and predictive molecular markers to better select patients who would benefit from a specific intervention is likely to be integral. Therefore, the possibility that irinotecan combined with LV and 5FU might be effective as adjuvant therapy in certain subsets of patients with colon cancer defined by various markers seems to be reasonable [6,25]. In this context, we have retrospectively analyzed by immunohistochemistry paraffin-embedded tumor tissues for detection of thymidylate synthase and topoisomerase I in patients treated with adjuvant chemotherapy within HeCOG protocols and we found that those expressing topoisomerase I seem to benefit from irinotecan-containing adjuvant chemotherapy [26]. Of particular interest is the CALGB 89803 study [20] that did not show, as previously mentioned, any differences in survival outcomes between IFL arm and LV plus 5FU arm. However, loss of tumor DNA mismatch repair (MMR) function could predict improved five-year DFS in patients treated with the IFL regimen as compared with those receiving LV plus 5FU [27]. Since the subset of patients who could derive a benefit from adjuvant irinotecan seems to represent a relatively small fraction, it is unlikely to be recognized when different populations are analyzed together. Finally, another speculation why irinotecan does not work in the adjuvant setting is the possibility that metastatic colon cancers may have different biological characteristics compared to primary tumors that could explain greater efficacy of irinotecan in metastatic disease.

## Conclusions

The results of our trial demonstrated that weekly bolus irinotecan plus LV plus 5FU should not be used in the adjuvant setting for colon cancer. Since the number of agents that are potentially effective in the systemic treatment of completely resected stage II or III disease is increasing, it is important to ascertain which subgroups of patients will benefit from a specific treatment.

## Abbreviations

ALT = alanine transaminase; AST = aspartate aminotransferase; CA = cancer antigen; CBC = complete blood cell; CEA = carcinoembryonic antigen; CNS = central nervous system; CPT-11 = irinotecan; CT scan = computed tomography; DFS = disease-free survival; ECG = electrocardiogram; FOLFIRI = 5fluorouracil, leucovorin and irinotecan; FOLFOX = 5fluorouracil, leucovorin and oxaliplatin; 5FU = 5-fluorouracil; HR = hazard rate; IFL regimen = irinotecan, 5fluorouracil and leucovorin; IV = intravenously; G-CSF = Granulocyte colony-stimulating factor; LV = leucovorin; MMR = mismatch repair; OS = overall survival; WHO = world health organization

## Competing interests

The authors declare that they have no competing interests.

## Authors' contributions

CAP conceived the idea, participated in the design of the study, contributed to the acquisition of data and the statistical analysis, and drafted the manuscript. PP conceived the idea, participated in the design of the study and contributed to the acquisition of data. LM substantially contributed to the statistical analysis and critically revised the manuscript. MK, GP, AB, GB, NX, GK, CK, TE, IK, NP and FM were involved in the acquisition of data (treatment of the patients with surgery and chemotherapy). DM, GK, IP and IE centrally evaluated all histology specimens. MAD, DB, ES, GA, DP, TM and HPK made substantial contributions in the conception and design of the study and the acquisition of data and critically revised the manuscript. GF made substantial contributions in the conception and design of the study and the acquisition of data and critically revised the manuscript. All authors read and approved the final manuscript.

## Pre-publication history

The pre-publication history for this paper can be accessed here:

http://www.biomedcentral.com/1741-7015/9/10/prepub
